# Protective effects on myelosuppression mice treated by three different classic Chinese medicine formulae

**DOI:** 10.4103/0973-1296.80671

**Published:** 2011

**Authors:** An-bin Zhao, Bin YU, Xian-Lin WU, Ke-Jian Cao, En-Qing LI, Qing-Mei LI, Xiao-Yin Chen

**Affiliations:** *Department of Internal Medicine, Xinzheng Chinese Medical Hospital, Zhengzhou, China*; 1 *Department of Traditional Chinese Medicine, Medical College, Jinan University, Guangzhou, People’s Republic of China*; 2 *Department of Traditional Chinese Medicine, School of Chinese Medicine, The University of Hong Kong, Hong Kong*; 3 *Department of Laboratory, Guangzhou Red Cross Hospital, Guangzhou, China*; * The first two authors contributed equally to this work

**Keywords:** Buzhong Yiqi decoction, cell cycle, compound Danshen decoction, Liuwei Dihuang decoction, myelosuppression, thrombopoietin, thrombopoietin receptor

## Abstract

**Background:**

In order to observe the protective therapeutic action and mechanism of Liuwei Dihuang Decoction, Buzhong Yiqi Decoction, and Compound Danshen Decoction on Myelosuppression induced by cyclophosphamide.

**Materials and Methods:**

The mice model was established by intraperitoneal injected with 100 mg/kg cyclophosphamide by human and mice dose conversion on the 9^th^, 11^th^, 13^th^ days during the experiment. Flow cytometry (FCM) was used for detecting the number of cells and investigating bone marrow cell cycles. Spleen was taken out and the mRNA expression level of thrombopoietin (TPO) and c-Mpl were detected by Q-PCR, and c-Mpl in spleen in order to discuss the mechanism of myelosuppression and the protective effects of traditional Chinese medicine.

**Results:**

Both Liuwei Dihuang Decoction Group and Buzhong Yiqi Decoction Group can accelerate bone marrow hematopoietic stem progenitor cells (HSPCs) in marrow-suppressed mice and enhance cell proliferation by promoting cell cycles from G0/G1 phase to access into S, G2/M phase. And at the same time these Chinese decoctions can increase the mRNA expression level of TPO and c-Mpl in spleen.

**Conclusion:**

Researched showed that Chinese formula take effect by affecting these genes on myelosuppressed mice.

## INTRODUCTION

Myelosuppression is a disease in the production of blood cells, and will induce fatigue, anemia, neutropenia, and/or thrombocytopenia. One of the common causes of myelosuppression is the side effects of cancer treatment, many of the drugs used in chemotherapy can induce myelosuppression. There are no effective methods to treat myelosuppression once it occurs, transfusions could be effective in replenishing red blood cells and platelets, and another alternative way is growth factor injections— include erythropoietin, granulocyte colony-stimulating factor (G-CSF, or filgrastim), granulocyte macrophage colony-stimulating factor (GM-CSF, or sargramostim), and interleukin 11 (oprelvekin), which can boost bone marrow performance,[1,2] but both of them can’t improve white blood cell level. Bone marrow transplant could be needed where bone marrow is damaged beyond self-repairing, but it is difficult to look for donators. In the past several years, lots of experimental studies on the effects of Chinese herbal medicines (CHM) to prevent and treat myelosuppression induced by chemotherapy have been carried out in China.[3]

Thrombopoietin (TPO) has been recently isolated as the ligand for the c-Mpl proto-onco-gene (Mpl-L). This hematopoietic growth factor appears as the homeostatic regulator of platelet production. Indeed, homozygous TPO or c-Mpl knockout mice exhibit a profound but not lethal thrombocytopenia.[4,5] *In vitro* and *in vivo* experiments have shown that TPO is the most potent growth factor for megakaryocyte (MK) differentiation, acting on different developmental stages, including MK progenitors, promegakaryoblasts, and MK. Experiments showed that daily TPO injection to animals, including mice and primates increased platelet count up to 10-fold the control value in a few days.[6]

The phenotypic alteration of c-Mpl^-^ and TPO^-^ deficient mice indicate a role of TPO in regulating hematopoietic progenitors. As expected, the primary phenotype of these mice is severe thrombocytopenia. The further detailed analysis of their marrow shows the mice with deficient progenitor pool as well.[5] Many clinical research work shows that the mutations of c-Mpl could be the cause of congenital amegakaryocytic thrombocytopenia.[7,8]

In this experiment, we used cyclophosphamide to establish myelosuppression mice model and then treated with traditional Chinese medicines. HSPC count and cycles were analyzed by flow cytometry (FCM). The mRNA expression level of TPO and c-Mpl were detected in order to investigate the mechanism of myelosuppression and the therapeutic effects of traditional Chinese medicines.

## MATERIALS AND METHODS

### Animals

One hundred male NH mice (specific pathogen free, 20–25g) were purchased from Laboratory Animal Center of Guangdong, certification No.0037530. The mice were used for the experiments after an acclimatization period of 6 days. Mice were given food and water throughout the experiments. The experimental protocol was approved by the local Ethics Review Committee for animal experimentation of Jinan University College of Medicine.

### Reagents

Hemolysin used in this experiment was purchased from Sysmex Shanghai Ltd. and Propidium Iodide (PI) from Sigma–Aldrich, Inc. (Shanghai, China). Cyclophosphamide (batch number. 08062621) from Jiangsu Hengrui Medicine Company (Jiangsu, China).

Antimouse CD34 and CD45 monoclonal antibodies were purchased from Biolengend Inc.(San Diego, CA, USA) and Trizol from Invitrogen Corp.(Invitrogen Guangzhou Office, China). cDNA reverse transcriptase kit from Bestbio Corp. (Shanghai, China) and Real-time Quantitative PCR kit from Genecopoeia Inc.(Rockville, MD, USA).

### Experimental drugs

Liuwei Dihuang Decoction: *Rehmannia glutinosa* 24 g, Common Yam Rhizome 12 g, Asiatic Cornelian Cherry Fruit 12 g, *Alisma orientalis* 9 g, *Poria cocos* 9 g, Cortex Moutan 9 g.

Buzhong Yiqi Decoction: Radix astragali preparata 30 g, *Radix codonopsitis* 15 g, Largehead Atractylodes 9 g, Angelica 9 g, *Cimicifuga foetida* 9 g, *Radix bupleuri* 9 g, Pericarpium Citri Reticulatae 9 g.

Compound Danshen Decoction: Salvia miltiorrhiza 30 g, sandalwood 9 g, Amomum villosum 9 g, safflower 9 g.

All of the Chinese medicines were purchased from the dispensary of traditional Chinese medicine in the first affiliated hospital of Jinan University. And 10 times distilled water was added into each prescription medicines and marinated for 30 min. Then the medicines were boiled for 30 mins, and the remainder added new distilled water and boiled for another hour. Then the medicine juice was strained, mixed, and concentrated to different consistencies, that is, Liuwei Dihuang Decoction 0.54 g/mL, Buzhong Yiqi Decoction 0.48 g/mL, and Compound Danshen Decoction 0.36 g/mL.

## METHODS

### Animal grouping

Mice were randomly divided into 5 groups, namely normal group (NR group), model group (MD group), Liuwei Dihuang Decoction group (LW group), Buzhong Yiqi Decoction group (BZ group), and Compound Danshen Decoction group (DS group), 20 in each group.

### Model establishment and treating

All the mice except in normal group were injected with 100 mg/kg cyclophosphamide intraperitoneally by human and mice body surface area dose conversion on the 9^th^, 11^th^, and 13^th^ days during the experiment. NR group and MD group were given 20 mL/kg distilled water, while other groups were given the same amount of Chinese medicines from the beginning to the end of the experiment. On the 14^th^ and 28^th^ day, 10 mice were killed in each group and the thigh bone and spleen were taken out for experimental detection.

### HSPC suspension preparation

Thigh bone was taken out under sterile conditions and marrow cavity was washed with saline to collect marrow cells with 200 wells filter. After that cell suspension was centrifuged at 2000g for 10 min, and 4ml 70% ethanol was added into deposit for fixation after supernatant was removed, and then oscillated to make HSPC suspension for next steps.

### HSPC count

Mouse IgG FITC (20 μL) and CD45 PE (20 μL) were added into control tubes, while CD34 FITC (20 μL) and CD45 PE (20 μL) were added into other group tubes. And then 50 μL HSPC suspension was added into each tube, incubated for 15 min and later mixed together and protected from light. After that, 2 mL hemolysin was added into of the each tubes and incubated for another 10 min after mixed together. Tubes were centrifuged at 3000g for 5 min and the supernatant was removed and 1 mL 1% PFA was added into tubes for HSPC count by FCM.

### HSPC cycle analysis

HSPC (2 mL) suspension was washed with PBS twice, and then PI dye was added for cell cycles detection. The percentage of cell phases was tested by FCM.

### Quantitative RT-PCR analysis

Spleen was taken out under sterile conditions and 100 mg spleen tissue was used for RNA extraction. Total RNA was isolated using Trizol and submitted to cDNA synthesis, after that, PCR amplification was carried out. The primers were designed based on the literatures and the cRNA sequence of TPO, c-Mpl, and beta-actin published by NCBI, and then ran agarose gel electrophoresis with the products of PCR amplification. Primer synthesized from Sangon Biology Company, Shanghai, China. Analysis was done using an aliquot of the reverse transcription reaction on the 7500 Fast Real-Time PCR System, with TPO, c-Mpl, beta-actin primers, and Sybr Green Master Mix. Data are presented as a relative expression levels normalized to beta-actin [Table 1].

**Table 1:** Primer sequences and products length

Gene	Primer sequence (5’–3’)	Products length (bp)
TPO	F: CCAGACGGAACAGAGCAAG	82
	R: CTGTCCTCGTGCTGCCAT	
c-Mpl	F: CCTGCACTGGAGGGAGGTCT	135
	R: GGCTCCAGCACCTTCCAGTC	
beta- Actin	F: CCAGCCTTCCTTCTTGGGTAT	97
	R: CATAGAGGTCTTTACGGATGTCAAC	

### Statistical analysis

All data were analyzed using Solutions Statistical Package for the Social Sciences 13.0.

Measurement data were compared with one-way analysis of variance, multiple groups means were compared with single factor analysis of variance, and the comparison among groups was performed with LSD method.

## RESULTS

### HSPC count

CD34 is a sialomucin molecule marker that is expressed on primitive hematopoietic stem cells and downregulated as they differentiate into mature cells. It is a cell surface glycoprotein and functions as a cell–cell adhesion factor. Although its precise function remains unknown, the pattern of expression of CD34 suggests that it plays a significant role in early hematopoiesis. So it is used as a surface marker for very early hematopoietic stem cells.

**Figure 1:**
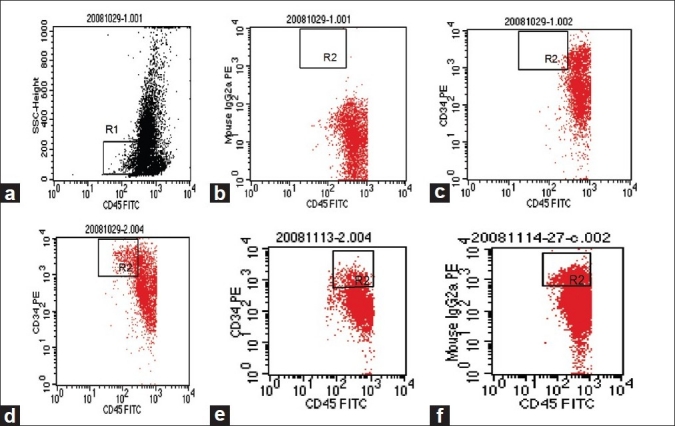
Hematopoietic stem progenitor cells count in each group by flow cytometry detection (a) Gate establishment (b) Negative control group (c) NR group (d) MD group (e) LW group (f) BZ group

Here we used FCM to detect the quantity of CD34. And the results were used for HSPC count [Figure 1].

On the 14^th^ day of the experiment, compared with NR group, the bone marrow hematopoietic stem and progenitor cells in the MD group were significantly decreased (2.76 ± 1.07, *P* < 0.01) [Table 2].

**Table 2:** Hematopoietic stem progenitor cells count in each group

Group	n	14^th^ day	28^th^ day
NR	10	3.69 ± 1.82	8.27 ± 4.50
MD	10	2.76 ± 1.07▲▲	4.95 ± 2.33▲
BZ	10	4.41 ± 1.55**	7.19 ± 3.89**
LW	10	3.46 ± 0.94*	8.05 ± 3.40**
DS	10	2.15 ± 0.94	5.87 ± 0.91**

▲ *P* < 0.05

▲▲ *P* < 0.01, compared with NR group

* *P* < 0.05

** *P* < 0.01, compared with MD group

Compared with MD group, bone marrow HSPCs were significantly increased in both LW group (3.46 ± 0.94, *P* < 0.05) and BZ group (4.41 ± 1.55, *P* < 0.01). On the 28^th^ day of the experiment, compared with NR group, the cell amount of bone marrow in the MD group was decreased statistically significantly (4.95 ± 2.33, *P* < 0.05); compared with the MD group, the cell amount of bone marrow was raised statistically significantly in BZ group, LW group, and DS decoction (7.19 ± 3.89, 8.05 ± 3.40, 5.87 ± 0.91, respectively, *P* < 0.01).

### Cell cycle analysis

On the 14^th^ day of the experiment, compared with NR group, the bone marrow hematopoietic stem and progenitor cells in G1 phase was increased (67.440 ± 8.4587, *P* < 0.05), S and G2 phase were significantly decreased (28.800 ± 9.8514, 2.490 ± 1.4910, *P* < 0.05).

Compared with MD group, FCM analysis showed that cells in G1 phase were significantly decreased in the other three groups (49.360 ± 6.8959, 52.410 ± 7.4445, 53.870 ± 10.0298, *P* < 0.05). However, increased in S and G2 phases with statistically significant difference (*P* < 0.05).

FCM data analysis showed that on the 28th day when compared with NR group, the cells in G1 phase were decreased significantly (72.940 ± 5.0943, *P* < 0.05), but cells in S and G2 phases were both increased (*P* < 0.05).

**Figure 2:**
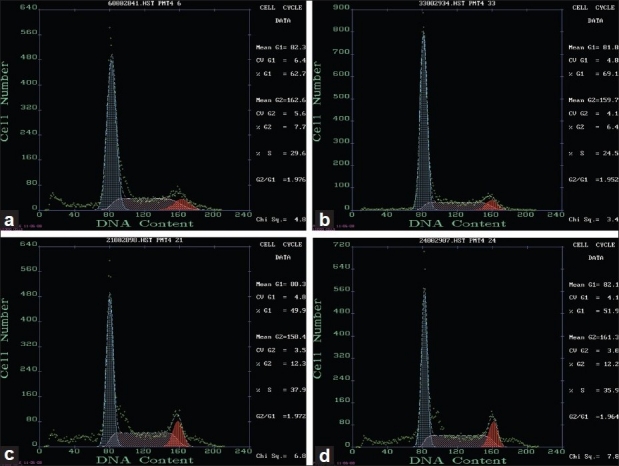
Cell cycle detection by flow cytometry on the 14^th^ day (a) NR group (b) MD group (c) BZ group (d) LW group

**Figure 3:**
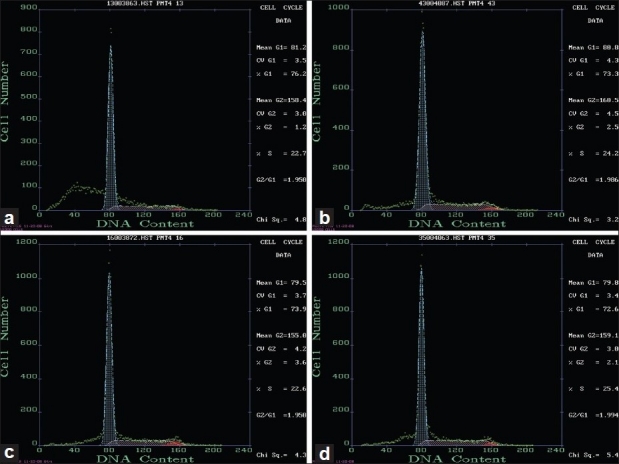
Cell cycle detection by flow cytometry on the 28^th^ day (a) NR group (b) MD group (c) BZ group (d) LW group

Compared with MD group, cell cycle analysis in BZ group, LW group, and DS group had no significant difference in G1, S, and G2 phases [Tables 3 and 4, Figures 2 and 3].

**Table 3:** Cell cycle analysis on the 14^th^ day

Group	n	G1 (%)	S (%)	G2 (%)
NR	20	64.250 ± 3.0843	32.773 ± 4.4371	4.700±1.0488
MD	20	67.440 ± 8.4587▲	28.800 ± 9.8514	2.490±1.4910▲
BZ	20	49.360 ± 6.8959**▲	43.850 ± 8.2199**▲▲	6.790±2.4520**▲
LW	20	52.410 ± 7.4445**▲	39.450 ± 10.2084*▲▲	6.920±2.6641**▲
DS	20	53.870 ± 10.0298*▲	36.676 ± 10.2056*▲	6.690±3.6483**▲

▲ *P*< 0.05

▲▲ *P* < 0.01, compared with NR group

* *P* < 0.05

** *P*< 0.01, compared with MD group

**Table 4:** Cell cycle analysis on the 28^th^ day

Group	n	G1 (%)	S (%)	G2 (%)
NR	10	76.850 ± 2.5295	21.867 ± 2.5544	1.810 ± 0.5174
MD	10	72.940 ± 5.0943▲	24.000 ± 4.1192▲	2.390 ± 1.1551▲
BZ	10	73.720 ± 3.1590▲	24.780 ± 3.7626▲	1.960 ± 0.9524
LW	10	72.940 ± 3.1053▲	25.940 ± 5.5580▲	1.760 ± 1.0265
DS	10	73.942 ± 3.7232▲	24.550 ± 4.0700▲	2.470 ± 0.8247▲

▲ *P*< 0.05

▲▲ *P* < 0.01, compared with NR group, *P < 0.05, **P < 0.01 compared with MD group,

### TPO and c-Mpl mRNA expression level

Quantitative RT-PCR results showed that on the 14^th^ and 28^th^ day of the experiment the TPO expression level was lower than that of the NR group with a significant difference (*P* < 0.05), and when compared with MD group, LW group and BZ group expressed more TPO mRNA with a significant difference (*P* < 0.05). But there were no significant differences on the expression level of TPO and c-Mpl mRNA either on the 14th or the 28th day of DS group [Table 5].

**Table 5:** Thrombopoietin mRNA expression level in each group

Group	n	14^th^ day	28^th^ day
NR	10	1.899 ± 0.505	2.089 ± 0.914
MD	10	0.465 ± 0.383▲▲	0.454 ± 0.237▲
BZ	10	2.583 ± 1.028**	3.146 ± 1.852*
LW	10	3.869 ± 1.552**	7.056 ± 1.611▲▲**
DS	10	1.259 ± 1.081	0.842 ± 0.324

▲ *P*< 0.05

▲▲ *P* < 0.01, compared with NR group

* *P* < 0.05

** *P* < 0.01, compared with MD group

There was no significant difference on the c-Mpl mRNA expression level when NR group was compared with model group during the experiment. Both LW group and BZ group increased the mRNA expression level with a significant difference (*P* < 0.05). But DS group had no effect on it [Table 6].

**Table 6:** C-Mpl mRNA expression level in each group

Group	n	14^th^ day	28^th^ day
NR	10	0.900 ± 0.312	0.671 ± 0.277
MD	10	0.842 ± 0.363	0.465 ± 0.366
BZ	10	3.192 ± 1.328▲*	4.397 ± 1.488▲▲**
LW	10	1.952 ± 0.499▲▲**	2.645 ± 1.393▲*
DS	10	1.207 ± 0.891	1.010 ± 0.490

▲ *P*< 0.05

▲▲ *P* < 0.01, compared with NR group

* *P* < 0.05

** *P* < 0.01, compared with MD group

## DISCUSSION

Currently, radiotherapy and chemotherapy used for treating cancer patients always cause bone marrow suppression, also known as myelosuppression, the main symptoms are the reduction of the number of blood cells, including white blood cells, red blood cells, and platelets. Most of this kind of patients with bone marrow suppression can develop low white blood cell counts, also known as leukopenia. Leukopenia can cause several symptoms, including fever, sore throat, and coughing, as well as shortness of breath. Chills, nasal congestion, and burning during urination may also occur with reduced amount of white blood cells. Individuals with insufficient amount of white blood cells are generally more susceptible to infections. Physicians may prescribe antibiotics to patients with this type of bone marrow suppression in order to fight off infections that are caused by bacteria. In recent years, some physicians treat leukopenia by granulocyte colony-stimulating factor (G-CSF) too, but it is reported that some solid tumor cells can secrete CSFs and/or expression of their receptors,[9] exogenous G-CSF receptor-positive CSFs may increase the rate of tumor metastasis and local recurrence rate,[10] so the security of G-CSF application is being questioned.

Our study was to explore the effects of Chinese herbs on bone marrow suppressed mice induced by chemotherapy. The myelosuppression mice model was established successfully, and proved by the tests of peripheral blood and bone marrow cells. Cyclophosphamide is a chemotherapeutic drug, it is metabolized in the body through the liver microsomal cytochrome P450 oxidation, the metabolites of phosphorus amide nitrogen mustard alkylating agent can induce cytotoxicity by influencing the formation of phosphorus amide nitrogen mustard and DNA cross-linking, inhibiting tumor cell growth and reproduction, but at the same time it can inhibit the rapid cell division of the normal tissues, such as the hematopoietic system. CD34 is a molecular marker; phosphorylation of CD34 glycoprotein mainly expresses in the early primary hematopoietic stem/progenitor cells, vascular endothelial cells, and embryonic fibroblast cells. In hematopoietic tissues, CD34 expression is highest in early hematopoietic progenitor cells, with cell differentiation and maturation. Therefore, detection of bone marrow CD34 cells may reflect the number of bone marrow hematopoietic stem cells.

Our study found that Liuwei Dihuang Decoction and Buzhong Yiqi Decoction can significantly increase hematopoietic stem cells numbers but Compound Danshen Decoction had no obvious effects.

The overwhelming majority cells in the bone marrow after receiving chemotherapy may stay in G1 phase and cannot pass G1/S check point.[11] Therefore, it is a hot area for antitumor treatment by driving cells from G1 phase into S phase and then enter G2/M phase. By using culture of bone marrow cells *in vitro* and FCM, Astragalus membranaceus injection may enhance hematopoietic function through promoting normal murine bone marrow cells (BMC) entering the proliferative cycle phase (S+M/G2).[12]

Another research found that Siwu Decoction could promote cells in the bone marrow from G0/G1 phase to S phase and then enter the next G2/M phase and recover the hematopoietic function in bone marrow mice.[13]

Our studies found that Liuwei Dihuang Decoction and Buzhong Yiqi Decoction, both of them can promote normal and myelosuppression cells into proliferation cycles. And also they can increase the rate of S+M/G2 cells, while decreasing G0/G1 phase cells and promoting cell proliferation.

From the results we can conclude that Liuwei Dihuang Decoction and Buzhong Yiqi Decoction may take effect by promoting cells in G2/M phase into G1 phase, and the decoctions could promote the production of DNA photolyase and repair damaged DNA after chemotherapy damage.

TPO is a ligand for c-Mpl that promotes both proliferation and differentiation of MKs *in vivo* and *in vitro*. Research data suggest that TPO may be involved in the abnormal proliferation and differentiation of human leukemic cells, especially of M7 and transient abnormal myelopoiesis (TAM) cells, considered to be of megakaryocytic lineage.[14]

The effectiveness of TPO in alleviating thrombocytopenia was initially evaluated in a placebo-controlled study involving rhesus monkeys exposed to 5-Gy total-body irradiation(TBI) using supraoptimal treatment with human recombinant TPO (10 μg/kg/d, subcutaneously, days 1–21 after TBI).[15,16] In addition, it has been shown that in animals TPO is able to accelerate platelet recovery after myeloablative chemotherapy. Therefore, TPO might be a potent therapeutic agent for life-threatening thrombocytopenic episodes associated with chemotherapy or bone marrow transplantation. TPO could also be a therapeutic agent in chronic thrombocytopenia due to platelet destruction. Indeed, it has been shown that TPO serum level is low in immune thrombocytopenic purpura (ITP). This may be due to the clearance of TPO by increased numbers of MK and young platelets.[17] In normal condition, c-Mpl expresses in MKs, platelets, and most of CD34 cells. TPO could enhance the formation and maturation of MKs.

TPO receptor, c-Mpl is a member of the hematopoietic growth factor receptor family, some experiments show a specific inhibition of MK colony formation by c-Mpl antisense oligonucleotide. The expression of c-Mpl is usually restricted to cells of megakaryocytic lineage, such as MKs, platelets, human leukemic cell lines with a megakaryocytic phenotype, and cells positive for CD34.[18]

Some studies have shown the c-Mpl gene is overexpressed at the mRNA level in acute myeloidleukemia (AML) and myelodysplastic syndrome. A recent study reported c-Mpl mRNA overexpression in 60% of a small sample of AML patients, and this overexpression correlates with shorter complete remission but not with karyotype group. These results suggest that c-Mpl protein overexpression in AML may play a role in the aggressiveness of this disease.[19]

In our experiment, we detected TPO and c-Mpl mRNA expression level in spleen, and found that the TPO mRNA expression level was significantly decreased when the myelosuppression model was established, but the level of c-Mpl mRNA had no significant difference compared with the normal group. Both Liuwei Dihuang Decoction Group and Buzhong Yiqi Decoction Group can increase the mRNA expression level of TPO and c-Mpl, but Compound Danshen Decoction had no significant impact on TPO and c-Mpl mRNA expression level during the experiment.

Why TPO mRNA expression level was decreased after model establishment, one of the reasons may be the spleen was damaged after treatment with cyclophosphamide, and induced many cell apoptosis. Another reason may be DNA repair, which can inhibit cells from translating genes into proteins before damaged tissues are repaired.

In our previous study,[20] Liuwei Dihuang Decoction, Buzhong Yiqi Decoction, and Compound Danshen Decoction could accelerate peripheral blood cells of the marrow-suppressed mice on the 14^th^ and 28^th^ day. However, in this experiment, Compound Danshen Decoction had no significant effects on both TPO and c-Mpl mRNA expression levels, which suggested that it takes effect through a different pathway and needs future research.
